# Numerical Investigation of the Dynamic Responses of Fibre-Reinforced Polymer Composite Bridge Beam Subjected to Moving Vehicle

**DOI:** 10.3390/polym14040812

**Published:** 2022-02-20

**Authors:** Eva Kormanikova, Kamila Kotrasova, Jozef Melcer, Veronika Valaskova

**Affiliations:** 1 Faculty of Civil Engineering, Technical University of Kosice, 042 00 Kosice, Slovakia; kamila.kotrasova@tuke.sk; 2 Faculty of Civil Engineering, University of Zilina, 010 08 Zilina, Slovakia; jozef.melcer@uniza.sk

**Keywords:** fibre-reinforced composite, laminate, bridge beam, moving load, vibration

## Abstract

In modern industry, heavy traditional materials are being substituted with light and strong fibre-reinforced polymer composite materials. Bridges and railroads made of composite laminates are considerably affected by traffic loads. Therefore, it is very important to analyse this effect which would find practical applications in engineering designs. This paper explains the theoretical formulation that governs the dynamic response of a composite beam subjected to a moving load. The governing equations for the dynamic effect on the laminated composite bridge beam are explained here. The main theories in the micro–macro modelling of composite laminates are also described in the paper. Within the macro modelling, the Classical Laminate and Shear Deformation Laminate Theory of beams are presented. The symmetric cross-ply laminated bridge, made of boron/epoxy is under consideration. The computational two-dimensional model of the vehicle is adopted. The governing equations for the dynamic effect on the laminated composite bridge beam are explained. The calculation of the time response of the bridge for the characteristic speeds of the vehicle is performed in the environment of the MATLAB software. The maximum dynamic magnification factor for the dynamic analysis of a composite beam is found.

## 1. Introduction

The use of polymers in composite materials in modern engineering applications has been increasing rapidly [1]. The rapid growth in manufacturing industries has led to the need for the betterment of materials in terms of strength, stiffness, density, and lower cost with improved sustainability [2,3]. Composite materials have emerged as one of the materials possessing such betterment in properties serving their potential in a variety of applications [4,5,6].

Furthermore, the better fatigue performance [7], creep performance [8], and durability [9] of fibre-reinforced resin composites in the service environment are also the main factors to be considered compared to traditional steel. Fibre-reinforced polymer composites have great potential to replace steel for bridge cables, underground oil extraction, and ocean engineering, owing to their light weight, high strength, and desirable corrosion and fatigue resistance.

In modern engineering, the heavy beams of traditional materials are gradually being substituted by fibre-reinforced polymer composite beams of lower weight and higher strength. These beams are often considered important elements of structures. Structures such as railroads and bridges are always under the action of dynamic moving loads caused by the moving vehicular traffic. Therefore, the analysis of a laminated composite beam under the action of moving loads would find many practical applications and is of valuable interest in engineering designs. The composite material for a specific application usually requires the use of angle-ply and unsymmetric laminates. Thus, bend–stretch, shear–stretch, and bend–twist couplings will be present in these laminates. The elementary or classical laminate theory (CLT) of beams assumes that the transverse shear strains are negligible and plane cross-sections before bending remain plane and normal to the axis of the beam after bending (Bernoulli–Euler beam theory). In the Bernoulli–Euler beam theory, the transverse deflection is assumed to be independent of coordinates *x*_2_, *x*_3_ of the cross-section. If the composite materials have a very low transverse shear modulus compared to their in-plane moduli, the CLT is inadequate for the analysis of dynamic response, and the shear deformation theory must be applied (Timoshenko beam theory). The first-order shear deformation theory accounted for a constant state of transverse shear stresses, but the transverse normal stress is often neglected. The most significant difference between the classical and first-order shear deformation theory is the effect of including transverse shear deformation.

There has not been much work completed on the study of composite beams under the action of moving loads, although several researchers have given theoretical [10,11,12,13], numerical [14,15,16], and experimental [17,18,19] analyses of traditional beams and plates under such conditions.

Considering the different size scales of the mechanical modelling of structure elements composed of fibre-reinforced composites, the micro, macro, and structural modelling levels must be considered. Within the microscale, the hybrid parallel approach to the homogenization of transport processes in masonry composites is described [20]. The nonlocal strain gradient nonlinear resonance of bi-directional functionally graded composite micro/nano-beams under periodic soft excitation is described in [21]. In the research in [22] was found a more intense shift of the first resonance frequency peak position to higher frequencies with increasing filler concentrations for HDPE/mica in comparison to HDPE/wollastonite composites. The research work [23] combines the scientific field of the micro–macro modelling of reinforced polymer composite laminates applied in the seismic response area of a rectangular composite tank filled with liquid. Frequency-dependent damped vibrations of multifunctional foam plates sandwiched and integrated by composite faces on the macroscale are explained in [24].

Fibre-reinforced polymer composites were originally developed for the aerospace and defence industries [25]. However, fibre-reinforced polymer composites have great potential for use in civil infrastructure [26]. One of their first uses was in an all-composite bridge superstructure in Miyun, China, in 1982 [27]. These materials are gradually gaining recognition from civil engineers as a new construction material [28]. Over the next few years, they proved successful in several areas of application: mainly in the form of sheets and strips for reinforcing existing bridge structures, as well as reinforcing bars, which replace the traditionally used steel reinforcement in concrete [29].

In this paper, a solution based on a CLT has been developed for the study of the dynamic response of an orthotropic laminated composite beam under the actions of moving loads [30,31,32,33]. The algorithm also accounts for the translatory and rotary inertia effects [34,35]. The differential equations are solved by numerical methods. The program MATLAB serves procedures for solving the differential equations of the first order by the Runge–Kutta–Fehlberg method [15]. The algorithm presented in this paper can be applied to moving loads with a constant-speed motion or constant-acceleration motion for the Bernoulli–Euler beam theory. A computer code has been developed for the analysis of orthotropic unsymmetric laminated composite beams under the action of moving loads.

## 2. Methods of Modelling and Analysis of Laminate Beam Theories

Summarizing the different size scales of the mechanical modelling of structure elements composed of fibre-reinforced composites, it must be noted that three modelling levels must be considered.

### 2.1. Microscale Level

**Figure 1:**
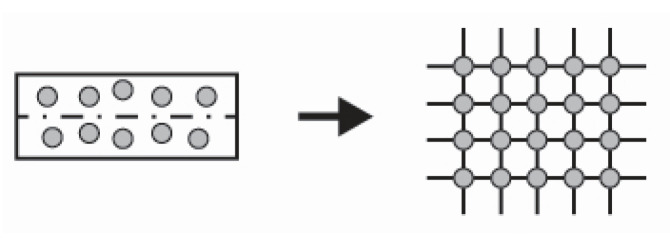
A periodic microstructure model for effective elastic properties of a bundle.

For a composite with a random microstructure, it is suitable to use the periodic microstructure model. The model for long cylindrical fibres, regularly arranged in a square microstructure, is illustrated in Figure 1.

In the last decade, effective media theories, widely used in classical continuum micromechanics, have been recognised as an attractive alternative to FE-based methods. Since its introduction, the Mori–Tanaka (MT) method [25] has enjoyed a considerable interest in a variety of engineering applications. The Mori–Tanaka method considers the effect of phase interactions on the local stresses by assuming an approximation in which the stress in each phase is equal to that of a single inclusion embedded into an unbounded matrix subjected to a yet unknown average matrix strain. 

The constitutive equation for unidirectional composite is written as

(1) $$\left\{\begin{array}{c} \sigma_{1}\\ \sigma_{2}\\ \sigma_{3}\\ \begin{array}{c} \sigma_{4}\\ \sigma_{5}\\ \sigma_{6} \end{array} \end{array}\right\}=\left[\begin{array}{cccccc} n & l & l & 0 & 0 & 0\\ l & \left(k+m\right) & \left(k-m\right) & 0 & 0 & 0\\ l & \left(k-m\right) & \left(k+m\right) & 0 & 0 & 0\\ 0 & 0 & 0 & m & 0 & 0\\ 0 & 0 & 0 & 0 & p & 0\\ 0 & 0 & 0 & 0 & 0 & p \end{array}\right]\left\{\begin{array}{c} \varepsilon_{1}\\ \varepsilon_{2}\\ \varepsilon_{3}\\ \varepsilon_{4}\\ \varepsilon_{5}\\ \varepsilon_{6} \end{array}\right\},$$

where:

(2) $$k=-{\left(1/G_{23}-4/E_{22}+4\nu_{12}^{2}/E_{11}\right)}^{-1},\;l=2k\nu_{12},\;m=G_{23},\;n=E_{11}+4k\nu_{12}^{2}=E_{11}+l^{2}/k,\;p=G_{12}.$$

From the application of the Mori–Tanaka method are obtained effective material characteristics at the microscale level, suitable for the 3D modelling of the composite structural element at the macroscale level.

**Figure 2:**
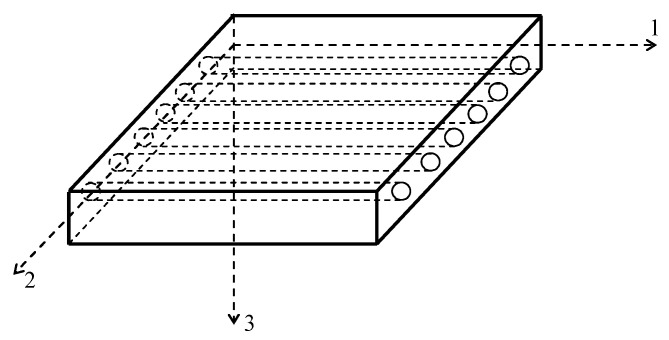
Unidirectional lamina with principal material axis *L* = 1 and *T* = 2 (on-axis).

A thin lamina is assumed to be under a state of plane stress. Two cases of material behaviour of laminae are of special interest for engineering applications: 

Long fibres with one unidirectional fibre orientation, the so-called unidirectional laminae, or UD-laminae, with loading along the material axis (on-axis case). The elastic behaviour of UD-laminae depends on the loading reference coordinate systems. In the on-axis case, the reference axes (1, 2) are identical to the material or principal axes of the lamina parallel and transverse to the fibre direction (Figure 2). The 1-axis is also denoted as *L*-axis and the 2-axis as *T*-axis (on-axis case). The elastic behaviour is macroscopically quasi-homogeneous and orthotropic with four independent material moduli $E_{1}\equiv E_{L}$, $E_{2}\equiv E_{T}$, $E_{6}\equiv G_{12}=G_{LT}$, and $\nu_{12}\equiv \nu_{LT}$.

The in-plane stress–strain relations are with

(3) $$E_{11}=\frac{E_{1}}{1-\nu_{12}.\nu_{21}}\;E_{22}=\frac{E_{2}}{1-\nu_{12}.\nu_{21}}\;E_{12}=\nu_{12}{\left(E_{22}\right)}_{L}\;E_{66}=G_{12}.$$

The relations of the in-plane stress components with the in-plane strain components are described by

(4) $$\sigma =E\varepsilon \Leftrightarrow \left(\begin{array}{c} \sigma_{11}\\ \sigma_{22}\\ \tau_{12} \end{array}\right)=\left(\begin{array}{ccc} E_{11} & E_{12} & 0\\ & E_{11} & 0\\ sym. & & E_{66} \end{array}\right)\left(\begin{array}{c} \varepsilon_{11}\\ \varepsilon_{22}\\ \gamma_{12} \end{array}\right)$$

UD-lamina with loading along the arbitrary axis (*x*_1_–*x*_2_) is different from the material axis (off-axis case). The elastic behaviour is macroscopically quasi-homogeneous and anisotropic. The in-plane stress–strain relations are formulated by fully populated matrices with all components different from zero, but the number of independent material constants is still four. 

**Figure 3:**
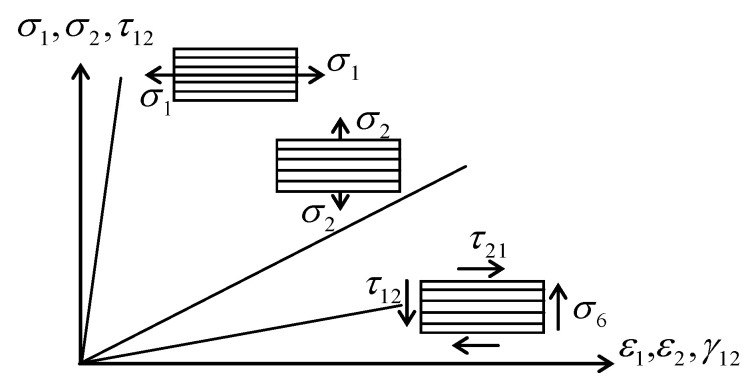
On-axis stress–strain for UD-lamina.

Figure 3 illustrates qualitatively the on-axis elastic behaviour of the UD-lamina [26].

The values *E_ij_* of the reduced stiffness matrix ***E*** depend on the effective moduli of the UD-lamina. These relations simplify the problem from a three-dimensional to a two-dimensional or plane stress state. For on-axis loading, the elastic behaviour is orthotropic with $E_{16}=E_{26}=0$, there is as in isotropic materials no coupling of normal stresses, and shear strains and shear stresses applied in the (*L*-*T*)-plane do not result in any normal strains in the *L* and *T* direction. The UD-lamina is therefore also called an especially orthotropic lamina.

A unidirectional lamina has very low stiffness and strength properties in the transverse direction compared with these properties in the longitudinal direction.

### 2.2. Mesoscale Level

**Figure 4:**
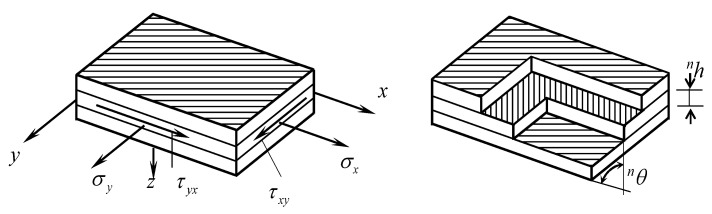
The global material reference system.

Laminates are constituted generally of different layers at different orientations. To study the elastic behaviour of laminates, it is necessary to take a global coordinate system for the whole laminate and to refer the elastic behaviour of each layer to this reference system. The global material reference system is given in Figure 4. 

We consider the ply material axes to be rotated away from the global axes by an angle θ, positive in the counterclockwise direction. This means that the (*x*_1_, *x*_2_)-axes are at an angle θ clockwise from the material axes. Thus, transformation relations are needed for the stresses, the strains, the stress–strain equations, the stiffness, and the compliance matrices. 

Note the relations for the transformation matrices

(5) En=T-T(θn)(n)ELT-(θn)

with transformation matrix for CLT

(6) $$T\left(\theta \right)=\left(\begin{array}{ccc} c^{2} & s^{2} & 2sc\\ s^{2} & c^{2} & -2sc\\ -sc & sc & c^{2}-s^{2} \end{array}\right)$$

and for shear deformation theory

(7) $$\hat{T}\left(\theta \right)=\left(\begin{array}{cc} T\left(\theta \right) & 0\\ 0^{T} & T^{t}\left(\theta \right) \end{array}\right)=\left(\begin{array}{ccccc} c^{2} & s^{2} & 2sc & 0 & 0\\ s^{2} & c^{2} & -2sc & 0 & 0\\ -sc & sc & c^{2}-s^{2} & 0 & 0\\ 0 & 0 & 0 & c & s\\ 0 & 0 & 0 & -s & c \end{array}\right)$$

where

(8) $$\overset{-}{T}\left(\theta \right)={\left(T^{T}\left(\theta \right)\right)}^{-1}$$

where *c* and *s* are noted as *s* = sin*θ* and *c* = cos*θ*.

In the theory of laminates, the most complex problem is the modelling and analysis of laminate with an arbitrary stacking of the layers. These laminates present couplings of stretching and bending, stretching and twisting, bending and twisting and the design engineer must look for simplifications. 

The first and most important simplification is to design symmetric laminates for which no coupling exists between the in-plane forces and flexural moments. The coupling terms *B_ij_* of the constitutive equations vanish. An additional simplification occurs when no bending–twisting coupling exists, i.e., the terms *D*_16_ and *D*_26_ are zero. Symmetric laminates for which no bending–twisting coupling exists are referred to as especially orthotropic laminates. Symmetrically balanced laminates with a great number of layers have an especially orthotropic behaviour. This class of laminates is greatly simplified and will be used to gain a basic understanding of laminate structural element response. 

The internal forces can be written in matrix form for CLT

(9) $$\left[\begin{array}{c} N_{x}\\ N_{y}\\ N_{xy} \end{array}\right]=\left[\begin{array}{ccc} A_{11} & A_{12} & A_{16}\\ A_{21} & A_{22} & A_{26}\\ A_{61} & A_{62} & A_{66} \end{array}\right]\left[\begin{array}{c} {\overset{-}{\varepsilon }}_{xx}^{}\\ {\overset{-}{\varepsilon }}_{yy}^{}\\ {\overset{-}{\gamma }}_{xy}^{} \end{array}\right]+\left[\begin{array}{ccc} B_{11} & B_{12} & B_{16}\\ B_{21} & B_{22} & B_{26}\\ B_{61} & B_{62} & B_{66} \end{array}\right]\left[\begin{array}{c} \kappa_{x}\\ \kappa_{y}\\ \kappa_{xy} \end{array}\right]$$

(10) $$\left[\begin{array}{c} M_{x}\\ M_{y}\\ M_{xy} \end{array}\right]=\left[\begin{array}{ccc} B_{11} & B_{12} & B_{16}\\ B_{21} & B_{22} & B_{26}\\ B_{61} & B_{62} & B_{66} \end{array}\right]\left[\begin{array}{c} {\overset{-}{\varepsilon }}_{xx}^{}\\ {\overset{-}{\varepsilon }}_{yy}^{}\\ {\overset{-}{\gamma }}_{xy}^{} \end{array}\right]+\left[\begin{array}{ccc} D_{11} & D_{12} & D_{61}\\ D_{21} & D_{22} & D_{26}\\ D_{61} & D_{62} & D_{66} \end{array}\right]\left[\begin{array}{c} \kappa_{x}\\ \kappa_{y}\\ \kappa_{xy} \end{array}\right]\;\;\;$$

and for shear deformation theory

(11) $$\left(\begin{array}{c} N\\ M\\ V \end{array}\right)=\left(\begin{array}{ccc} A & B & 0\\ B & D & 0\\ 0 & 0 & \overset{-}{A} \end{array}\right)\left(\begin{array}{c} \overset{-}{\varepsilon }\\ \kappa \\ \gamma \end{array}\right)$$

### 2.3. Macroscale Level

On the macroscale or structural level, the mechanical response of structural members such as beams, plates, shells, etc. must be analysed taking into account possibilities to formulate structural theories of a different order. 

#### 2.3.1. Classical Laminate Beam Theory

Frequently, as engineers try to optimise the use of materials, they design composite beams made from two or more materials. The design rationale is quite straightforward. For bending loading, stiff, strong, heavy, or expensive material must be far away from the neutral axis at places where its effect will be greatest. The weaker, lighter, or less expensive material as composite laminates will be used in the part of the bridge beam under the moving load. Laminate beams with simple or double symmetric cross-sections are most important in engineering applications. The derivations are therefore limited to straight beams with simple symmetric constant cross-sections. The bending moments act in a plane of symmetry. Also, cross-sections consisting of partition walls and orthogonal to the plane of bending are considered. We consider elementary beam equations. The cross-section area can have various geometries but must be symmetric to the *z*-axis. 

The known equations for the stress

(12) $$\sigma \left(x,z\right)=\overset{-}{\varepsilon }\left(x\right)E\left(z\right)+z\kappa \left(x\right)E\left(z\right)$$

where *E = E_eff_* is the effective elastic modulus in longitudinal direction.

The strains are deduced from the displacements

(13) $$\varepsilon \left(x,z\right)=\overset{-}{\varepsilon }\left(x\right)+z\kappa \left(x\right)$$

with

$$\overset{-}{\varepsilon }=\frac{\partial \overset{-}{u}}{\partial x}$$

and

(14) $$\;\kappa =-\frac{\partial \psi }{\partial x}$$

Then strain ε can be written as

(15) $$\;\kappa =-\frac{\partial \psi }{\partial x}$$

The internal forces *N*, *M* can be obtained from

(16) $$N=\int_{-h/2}^{+h/2}E\left(z\right)dz\overset{-}{\varepsilon }+\int_{-h/2}^{+h/2}E\left(z\right)zdz\kappa \;M=\int_{-h/2}^{+h/2}E\left(z\right)zdz\overset{-}{\varepsilon }+\int_{-h/2}^{+h/2}E\left(z\right)z^{2}dz\kappa$$

with stretching, coupling, and bending shear stiffness:

(17) A=b∫−h/2+h/2E(z)dz=b∑n=1N∫zn−1znEndz=b∑n=1NEnnhB=b∫−h/2+h/2E(z)zdz=b∑n=1N∫zn−1znEnzdz=b∑n=1NEnzn2−zn−122D=b∫−h/2+h/2E(z)z2dz=b∑n=1N∫zn−1znEnz2dz=b∑n=1NEnzn3−zn−133

where *b* is the beam width, *^n^E* is the effective elastic modulus in the longitudinal direction of *n*th layer and *A* = *bA*_11_, *B* = *bB*_11_, *D* = *bD*_11_.

The constitutive equation can by written in the condensed matrix form:

(18) $$\left(\begin{array}{c} N\\ M \end{array}\right)=\left(\begin{array}{cc} A & B\\ B & D \end{array}\right)\left(\begin{array}{c} \overset{-}{\varepsilon }\\ \kappa \end{array}\right)$$

#### 2.3.2. Shear Deformation Laminate Beam Theory

The classical laminate theory allows us to calculate the stresses and strains with high precision for very thin laminates except in a little extended region near the free edges. The validity of the classical theory has been established by comparing theoretical results with experimental tests and with more exact solutions based on the general equations of the linear anisotropic elasticity theory. If the width-to-thickness ratio is less than 20, the results derived from the classical theory show significant differences with the actual mechanical behaviour and the modelling must be improved. A first improvement is to the effect of shear deformation in the framework of a first-order displacement approach. A further improvement is possible by introducing correction factors for the transverse shear moduli.

The known equations for the stresses are the following:

(19) $$\sigma \left(x,z\right)=\overset{-}{\varepsilon }\left(x\right)E\left(z\right)+z\kappa \left(x\right)E\left(z\right)\;\tau \left(x,z\right)=\gamma G\left(z\right)$$

where *E = E_eff_* and *G = G_eff_* are the effective elastic modulus in longitudinal direction and transversal direction.

The strains are deduced from the displacements

(20) $$\varepsilon \left(x,z\right)=\overset{-}{\varepsilon }\left(x\right)+z\kappa \left(x\right)\;\gamma \left(x,z\right)=\left(\frac{\partial \overset{-}{w\left(x\right)}}{\partial x}-\psi \left(x\right)\right)$$

with

$$\overset{-}{\varepsilon }=\frac{\partial \overset{-}{u}}{\partial x}$$

and

(21) $$\kappa =-\frac{\partial \psi }{\partial x}$$

Then strains can be written as

(22) $$\varepsilon =\frac{\partial u}{\partial x}=\frac{\partial \overset{-}{u}}{\partial x}-z\frac{\partial \psi }{\partial x}\;\gamma =\frac{\partial w}{\partial x}+\frac{\partial u}{\partial z}=\frac{\partial \overset{-}{w}}{\partial x}-\psi$$

The stress *σ_x_* *=* *σ*, varies linearly through a layer thickness and the stress *τ_xz_* *=* *τ* is constant through the thickness. There is no stress continuity through the laminate thickness but stress jumps from ply to ply at their interfaces depending on the reduced stiffness.

Internal forces can be written as

(23) $$N=\int_{-h/2}^{+h/2}E\left(z\right)dz\overset{-}{\varepsilon }+\int_{-h/2}^{+h/2}E\left(z\right)zdz\kappa \;M=\int_{-h/2}^{+h/2}E\left(z\right)zdz\overset{-}{\varepsilon }+\int_{-h/2}^{+h/2}E\left(z\right)z^{2}dz\kappa \;V=\left(k^{*}\right)\int_{-h/2}^{+h/2}E^{t}\left(z\right)dz\gamma$$

With stretching, coupling, and bending stiffnesses the same as in Equation (17). 

Transverse shear stiffness can be written as

(24) A-=b∫−h/2+h/2Et(z)dz=b∑n=1NEntnh

where *^n^E^t^* is the effective elastic modulus in the transversal direction of *n*th layer.

The constitutive equation can by written in the condensed matrix form:

(25) $$\left(\begin{array}{c} N\\ M\\ V \end{array}\right)=\left(\begin{array}{ccc} A & B & 0\\ B & D & 0\\ 0 & 0 & \overset{-}{A} \end{array}\right)\left(\begin{array}{c} \overset{-}{\varepsilon }\\ \kappa \\ \gamma \end{array}\right).$$

The shear stiffness values can be improved with help of shear correction factors. In this case, the part of the constitutive equation relating to the resultants *N*, *M* is not modified. The other part relating to transverse shear resultants *V* is modified by replacing the stiffness $\overset{-}{A}$ by k^*^$\overset{-}{A}$.

The parameters $k^{*}$ is the shear correction factor. A very simply approach is to introduce a weighting function *f*(*z*) for the distribution of the transverse shear stress through the thickness *h*.

Assume a parabolic function

(26) $$f\left(z\right)=\frac{5}{4}\left(1-{\left(\frac{z}{h/2}\right)}^{2}\right).$$

The transverse shear force is

(27) Vxz=∑n=1N∫hnτnxzf(z)dzVxz=54(∑n=1NEntγxz∫hn(1−(zh/2)2)dz)

The constitutive equation for shear force is

(28) $$V_{xz}=\overset{-}{A}\gamma_{xz}$$

For the components of $\overset{-}{A}$ gilt

(29) A-=54∑n=1NEnt((zn−zn−1)−43h2(zn3−zn−13))

This approach yields for the case of a single homogenous layer with $E^{t}=$*G_eff_* and shear correction factor $k^{*}=5/6$ for the shear stiffness.

The first-order shear deformation theory yields mostly sufficiently accurate results for the displacements and for the in-plane stresses.

For a midplane symmetric laminated composite beam subjected to a lateral load *p*_3_ and including transverse shear deformation:

(30) $$D\frac{\partial^{2}\psi }{\partial x^{2}}-k^{*}\overset{-}{A}\left(\psi +\frac{\partial w}{\partial x}\right)=0\;k^{*}\overset{-}{A}\left(\frac{\partial \psi }{\partial x}+\frac{\partial^{2}w}{\partial x^{2}}\right)+p_{3}=0$$

More often products and structures are subjected to vehicular dynamic loads. In the linear-elastic range, the dynamic effects can be divided into two categories: free vibrations and forced vibrations, and the latter can be further subdivided into one-time events or receiving loads. Free vibration problems are called eigenvalue problems. They are represented by homogeneous equations, for which nontrivial solutions only occur at certain characteristic values of a parameter, from which the natural frequencies are determined. In the general case of forced vibrations, the displacements, the rotations, and the transverse load *p*_3_ are functions of *x* and *t*. In-plane loading is not considered but in-plane displacement, rotary, and coupling inertia terms must be considered. 

The governing equations for the calculation of natural frequencies of especially orthotropic beams are

(31) $$D\frac{\partial^{2}\psi }{\partial x^{2}}-k^{*}\overset{-}{A}\left(\psi +\frac{\partial w}{\partial x}\right)-I\frac{\partial^{2}\psi }{\partial t^{2}}=0$$

(32) $$k^{*}\overset{-}{A}\left(\frac{\partial \psi }{\partial x}+\frac{\partial^{2}w}{\partial x^{2}}\right)-\rho_{m}h\frac{\partial^{2}w}{\partial t^{2}}=0$$

For the force vibration analysis of laminate beam gilt:

(33) $$k^{*}\overset{-}{A}\left(\frac{\partial \psi }{\partial x}+\frac{\partial^{2}w}{\partial x^{2}}\right)-\rho_{m}h\frac{\partial^{2}w}{\partial t^{2}}+p_{3}\left(x,t\right)=0$$

where

ρm=b∑n=1Nρn(zn−zn−1)

and

(34)  I=b13∑k=1Nρn(zn3−zn−13).

The terms involving $\rho_{m}$ and *I* are called translatory or rotatory inertia terms.

## 3. Bridge under Consideration, Discussion

**Figure 5:**
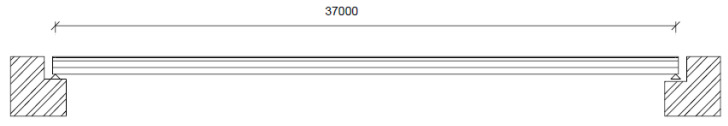
Simply supported bridge.

**Figure 6:**
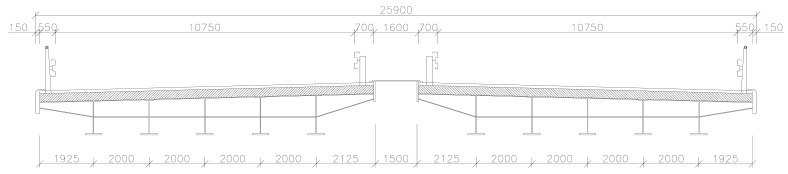
Transverse section of the bridge.

**Figure 7:**
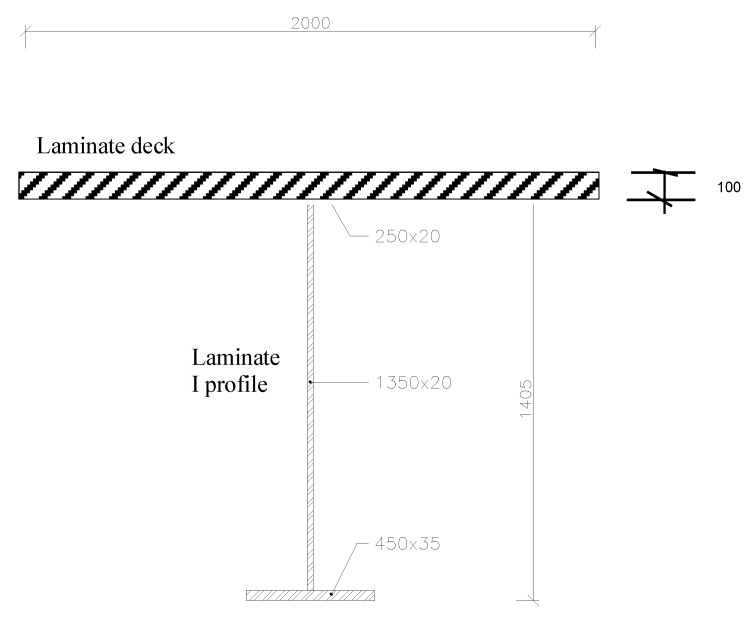
Transverse section of the one segment.

The symmetric cross-ply laminated bridge, ((0/90)_25_)_s_, made of boron/epoxy is analysed next, in Figure 5, Figure 6 and Figure 7. These are two separate bridges situated next to each other. The subject of the analysis is only one bridge. The geometric and material properties of this model are listed in Table 1.

### 3.1. A Computational Model of Vehicle and Bridge

**Figure 8:**
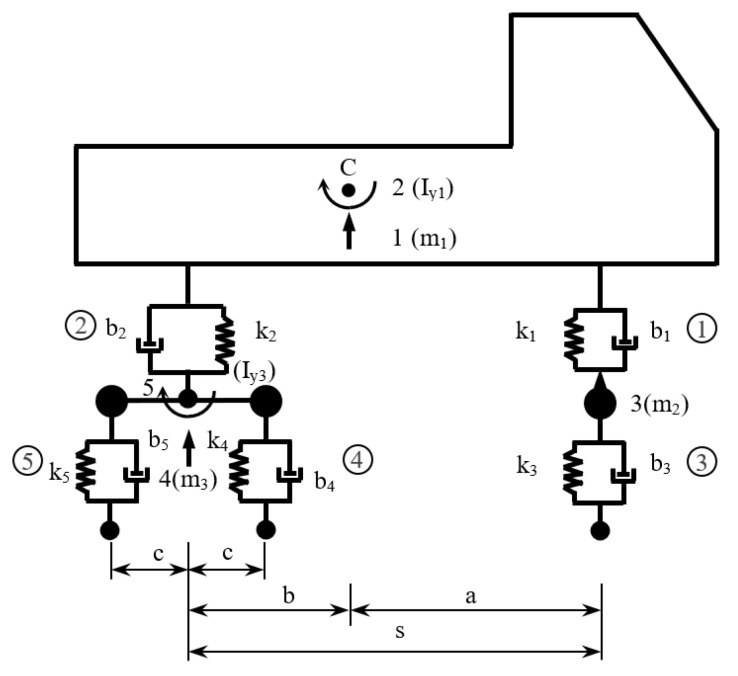
2D multi-body model of vehicle.

To solve the task of the bridge response to the effects of moving loads due to a heavy truck, a two-dimensional (2D) model was adopted in this case [10]. The model of the vehicle is a 2D multi-body model with eight degrees of freedom, Figure 8.

Five degrees of freedom are mass (displacements *r_i_*, *i* =1, 2, 3, 4, 5) and three are massless (displacements *v_i_*, *i* = 3, 4, 5). The displacement components corresponding to the degrees of freedom are arranged in the vector **{*r*}**.

(35) $$\left\{r\right\}={\left[r_{1}\left(t\right),r_{2}\left(t\right),r_{3}\left(t\right),r_{4}\left(t\right),r_{5}\left(t\right),v_{3}\left(t\right),v_{4}\left(t\right),v_{5}\left(t\right)\right]}^{T}$$

The relationship between the displacement components {r(t)} corresponding to the degrees of freedom and the deformations of the connecting members {d(t)} is presented by a transposed static matrix ${\left[A\right]}^{T}$ according to the relation

(36) $$\left\{d\left(t\right)\right\}={\left[A\right]}^{T}\cdot \left\{r\left(t\right)\right\}.$$

The dependence between the elastic forces in the connecting members (in terms of the action of mass objects on the connecting members) and their deformations is described by the equation

(37) $$\left\{F_{re}\left(t\right)\right\}=\left[k\right]\cdot \left\{d\left(t\right)\right\},$$

where [k] is the stiffness matrix of the connecting members. The dependence of the damping forces on the strain rate $\left\{\dot{d}\left(t\right)\right\}$ is

(38) $$\left\{F_{d}\left(t\right)\right\}=\left[b\right]\cdot \left\{\dot{d}\left(t\right)\right\}.\;$$

The resulting forces in the connecting members when acting on mass objects are

(39) $$\left\{F_{CM}\left(t\right)\right\}=-\left(\left\{F_{re}\left(t\right)\right\}+\left\{F_{d}\left(t\right)\right\}\right).\;$$

The sign (-) is a consequence of the principle of action and reaction. From the forces in the connecting members$\;\left\{F_{CM}\left(t\right)\right\}$, the static equivalents corresponding to the individual degrees of freedom$\;\left\{F_{DF}\left(t\right)\right\}$ are calculated according to the relation

(40) $$\left\{F_{DF}\left(t\right)\right\}=\left[A\right]\cdot \left\{F_{CM}\left(t\right)\right\}.$$

To the forces corresponding to the individual degrees of freedom$\;\left\{F_{DF}\left(t\right)\right\}$ it is necessary to add the gravitational forces$\;\left\{F_{G}\left(t\right)\right\}$ and reactions at the point of contact$\;\left\{F_{RC}\left(t\right)\right\}$, which gives a complete vector of forces acting on the given computational model $\left\{F_{R}\left(t\right)\right\}$

(41) $$\left\{F_{R}\left(t\right)\right\}=\left\{F_{DF}\left(t\right)\right\}+\left\{F_{G}\left(t\right)\right\}+\left\{F_{RC}\left(t\right)\right\}.$$

The system of equations describing the conditions of force equilibrium of the model has the form

(42) $$\left[m\right]\cdot \left\{\ddot{r}\left(t\right)\right\}=\left\{F_{R}\left(t\right)\right\},$$

where [m] is the mass matrix of the model. Derivations according to time *t* are indicated by a dot above the sign of the dependent variable. System (42) is finally subdivided into equations of motion describing the vibration of the vehicle (43) and the equations for calculating the reactions at the contact points (44)

(43) $$\begin{array}{ll} {\ddot{r}}_{1}\left(t\right)= & -\{b_{1}\cdot \left[{\dot{r}}_{1}\left(t\right)-a\cdot {\dot{r}}_{2}\left(t\right)-{\dot{r}}_{3}\left(t\right)\right]+b_{2}\cdot \left[{\dot{r}}_{1}\left(t\right)+b\cdot {\dot{r}}_{2}\left(t\right)-{\dot{r}}_{4}\left(t\right)\right]+k_{1}\cdot \left[r_{1}\left(t\right)-a\cdot r_{2}\left(t\right)-r_{3}\left(t\right)\right]+\\ & +k_{2}\cdot \left[r_{1}\left(t\right)+b\cdot r_{2}\left(t\right)-r_{4}\left(t\right)\right]\}/m_{1}, \end{array}\;\begin{array}{ll} {\ddot{r}}_{2}\left(t\right)= & -\{-a\cdot b_{1}\cdot \left[{\dot{r}}_{1}\left(t\right)-a\cdot {\dot{r}}_{2}\left(t\right)-{\dot{r}}_{3}\left(t\right)\right]+b\cdot b_{2}\cdot \left[{\dot{r}}_{1}\left(t\right)+b\cdot {\dot{r}}_{2}\left(t\right)-{\dot{r}}_{4}\left(t\right)\right]-\\ & -a\cdot k_{1}\cdot \left[r_{1}\left(t\right)-a\cdot r_{2}\left(t\right)-r_{3}\left(t\right)\right]+b\cdot k_{2}\cdot \left[r_{1}\left(t\right)+b\cdot r_{2}\left(t\right)-r_{4}\left(t\right)\right]\}/I_{y1}, \end{array}\;{\ddot{r}}_{3}\left(t\right)=-\{-b_{1}\cdot \left[{\dot{r}}_{1}\left(t\right)-a\cdot {\dot{r}}_{2}\left(t\right)-{\dot{r}}_{3}\left(t\right)\right]+b_{3}\cdot \left[{\dot{r}}_{3}\left(t\right)-{\dot{v}}_{3}\left(t\right)\right]-k_{1}\cdot \left[r_{1}\left(t\right)-a\cdot r_{2}\left(t\right)-r_{3}\left(t\right)\right]+\;\;\;\;\;\;\;\;+k_{3}\cdot \left[r_{3}\left(t\right)-v_{3}\left(t\right)\right]\}/m_{2},\;{\ddot{r}}_{4}\left(t\right)=-\{-b_{2}\cdot \left[{\dot{r}}_{1}\left(t\right)+b\cdot {\dot{r}}_{2}\left(t\right)-{\dot{r}}_{4}\left(t\right)\right]+b_{4}\cdot \left[{\dot{r}}_{4}\left(t\right)-c\cdot {\dot{r}}_{5}\left(t\right)-{\dot{v}}_{4}\left(t\right)\right]+b_{5}\cdot \left[{\dot{r}}_{4}\left(t\right)+c\cdot {\dot{r}}_{5}\left(t\right)-{\dot{v}}_{5}\left(t\right)\right]-\;\;\;\;-k_{2}\cdot \left[r_{1}\left(t\right)+b\cdot r_{2}\left(t\right)-r_{4}\left(t\right)\right]+k_{4}\cdot \left[r_{4}\left(t\right)-c\cdot r_{5}\left(t\right)-v_{4}\left(t\right)\right]+k_{5}\cdot \left[r_{4}\left(t\right)+c\cdot r_{5}\left(t\right)-v_{5}\left(t\right)\right]\}/m_{3},\;\;{\ddot{r}}_{5}\left(t\right)=-\{-c\cdot b_{4}\cdot \left[{\dot{r}}_{4}\left(t\right)-c\cdot {\dot{r}}_{5}\left(t\right)-{\dot{v}}_{4}\left(t\right)\right]+c\cdot b_{5}\cdot \left[{\dot{r}}_{4}\left(t\right)+c\cdot {\dot{r}}_{5}\left(t\right)-{\dot{v}}_{5}\left(t\right)\right]-\;\;\;\;\;\;\;\;-c\cdot k_{4}\cdot \left[r_{4}\left(t\right)-c\cdot r_{5}\left(t\right)-v_{4}\left(t\right)\right]+c\cdot k_{5}\cdot \left[r_{4}\left(t\right)+c\cdot r_{5}\left(t\right)-v_{5}\left(t\right)\right]\}/I_{y3},$$

(44) $$F_{RC,3}\left(t\right)=G_{3}-k_{3}\cdot \left[r_{3}\left(t\right)-v_{3}\left(t\right)\right]-b_{3}\cdot \left[{\dot{r}}_{3}\left(t\right)-{\dot{v}}_{3}\left(t\right)\right]=\;\;\;\;\;\;\;\;\;\;\;\;=g\cdot \left(m_{1}\cdot b/s+m_{2}\right)-k_{3}\cdot \left[r_{3}\left(t\right)-v_{3}\left(t\right)\right]-b_{3}\cdot \left[{\dot{r}}_{3}\left(t\right)-{\dot{v}}_{3}\left(t\right)\right],\;F_{RC,4}\left(t\right)=G_{4}-k_{4}\cdot \left[r_{4}\left(t\right)-c\cdot r_{5}\left(t\right)-v_{4}\left(t\right)\right]-b_{4}\cdot \left[{\dot{r}}_{4}\left(t\right)-c\cdot {\dot{r}}_{5}\left(t\right)-{\dot{v}}_{4}\left(t\right)\right]=\;\;\;\;\;\;\;\;=0,5\cdot g\cdot \left(m_{1}\cdot a/s+m_{3}\right)-k_{4}\cdot \left[r_{4}\left(t\right)-c\cdot r_{5}\left(t\right)-v_{4}\left(t\right)\right]-b_{4}\cdot \left[{\dot{r}}_{4}\left(t\right)-c\cdot {\dot{r}}_{5}\left(t\right)-{\dot{v}}_{4}\left(t\right)\right],\;F_{RC,5}\left(t\right)=G_{5}-k_{5}\cdot \left[r_{4}\left(t\right)+c\cdot r_{5}\left(t\right)-v_{5}\left(t\right)\right]-b_{5}\cdot \left[{\dot{r}}_{4}\left(t\right)+c\cdot {\dot{r}}_{5}\left(t\right)-{\dot{v}}_{5}\left(t\right)\right]=\;\;\;\;\;\;\;\;\;\;\;\;\;\;=0,5\cdot g\cdot \left(m_{1}\cdot a/s+m_{3}\right)-k_{5}\cdot \left[r_{4}\left(t\right)+c\cdot r_{5}\left(t\right)-v_{5}\left(t\right)\right]-b_{5}\cdot \left[{\dot{r}}_{4}\left(t\right)+c\cdot {\dot{r}}_{5}\left(t\right)-{\dot{v}}_{5}\left(t\right)\right].$$

The bridge is modelled as simple supported Bernoulli–Euler beam with continuously distributed mass. The equation of motion has the form

(45) $$E\cdot I\frac{\partial^{4}y\left(x,t\right)}{\partial x^{4}}+\mu \frac{\partial^{2}y\left(x,t\right)}{\partial t^{2}}+2\cdot \mu \cdot \omega_{b}\frac{\partial \;y\left(x,t\right)}{\partial t^{}}=p\left(x,t\right)$$

where *E* is modulus of elasticity, *I* is quadratic moment of cross section, *μ* is mass intensity per meter of length, *ω_b_* angular damping frequency, *y*(*x*,*t*) is dynamic bending line, and *p*(*x*,*t*) is the intensity of the continuous distributed load. Equation (45) is a partial differential equation. Since the equations of motion of the vehicle are ordinary differential equations, it is also appropriate to transform the bridge equation of motion into an ordinary differential equation. This can be performed by assuming the shape of the bending line of the bridge *y*_0_(*x*)

(46) $$y\left(x,t\right)=q\left(t\right)\cdot y_{0}\left(x\right)=q\left(t\right)\cdot \sin\frac{\pi \cdot x}{L}.$$

The proportionality coefficient *q*(*t*) has the meaning of the generalised Lagrange coordinate and *L* is the span of the bridge. Substituting assumption (46) into equation (45) produces

(47) $$\left\{\ddot{q}\left(t\right)\cdot \mu +\dot{q}\left(t\right)\cdot 2\cdot \mu \cdot \omega_{b}+q\left(t\right)\cdot E\cdot I\frac{\pi^{4}}{L^{4}}\right\}\sin\frac{\pi .x}{L}=p\left(x,t\right).$$

In the case of motion of discrete forces *F_j_*, the continuously distributed load *p*(*x*,*t*) can be expressed by the use of Dirac function [8] in the form

(48) $$p\left(x,t\right)=\sum_{j}\varepsilon_{j}\cdot \delta \left(x-x_{j}\right)\cdot F_{j}\left(t\right)=\sum_{j}\sum_{n=1}^{\infty }p_{n,j}\left(t\right)\cdot \sin\frac{n\cdot \pi \cdot x}{L},$$

where

(49) $$p_{n,j}\left(t\right)=\frac{2}{L}\int_{0}^{1}p_{j}\left(x,t\right)\cdot \sin\frac{n.\pi .x}{L}dx=\frac{2}{L}\varepsilon_{j}\cdot F_{j}\left(t\right)\cdot \sin\frac{n\cdot \pi \cdot x_{j}}{L}.$$

Then

(50) $$\begin{array}{ll} p\left(x,t\right) & =\underset{j}{\sum }\overset{\infty }{\underset{n=1}{\sum }}p_{n,j}\left(t\right)\cdot \sin\frac{n\cdot \pi \cdot x}{L}=\underset{j}{\sum }\overset{\infty }{\underset{n=1}{\sum }}\frac{2}{L}\varepsilon_{j}\cdot F_{j}\left(t\right)\cdot \sin\frac{n\cdot \pi \cdot x_{j}}{L}\cdot \sin\frac{n\cdot \pi \cdot x}{L}=\\ & =\sum_{j}\sum_{n=1}^{\infty }\frac{2}{L}\varepsilon_{j}\cdot F_{\mathrm{int},j}\left(t\right)\cdot \sin\frac{n.\pi .x_{j}}{L}\cdot \sin\frac{n\cdot \pi \cdot x}{L}. \end{array}$$

If we take into account only the first member of the series, the expression takes the form

(51) $$p\left(x,t\right)=\frac{2}{L}\sin\frac{\pi \cdot x}{L}\sum_{j}\varepsilon_{j}\cdot F_{j}\left(t\right)\cdot \sin\frac{\pi \cdot x_{j}}{L}.$$

If the force *F_j_* is already on the beam, then *ε_j_* = 1, if *F_j_* is outside the beam, *ε_j_* = 0. The time *t* = 0 corresponds to the input of the first axle on the beam. If we are looking for a response only in the middle of the bridge span, *x* = *L*/2, then sin(π⋅x/L)=1, and the equation of motion of the bridge takes the form

(52) $$\ddot{q}\left(t\right)\cdot \mu +\dot{q}\left(t\right)\cdot 2\cdot \mu \cdot \omega_{b}+q\left(t\right)\cdot E\cdot I\frac{\pi^{4}}{L^{4}}=\frac{2}{L}\underset{j}{\sum }\varepsilon_{j}\cdot F_{j}\left(t\right)\cdot \sin\frac{\pi \cdot x_{j}}{L}.$$

It is possible to work with the unevenness of the road *u*(*t*) and define the resulting profile of the road surface as

(53) v(x,t)=y(x,t)+u(t).

### 3.2. Results of Numerical Solution

The numerical solution of the equations of motion was performed in the environment of MATLAB system [12]. The fourth order Runge–Kutta method was used (ode45 procedure). The second order differential equations were transformed to the first order equations by the following substitution

(54) $$r_{1}\left(t\right)=y_{1}\left(t\right),\;r_{2}\left(t\right)=y_{3}\left(t\right),\;r_{3}\left(t\right)=y_{5}\left(t\right),\;r_{4}\left(t\right)=y_{7}\left(t\right),\;r_{5}\left(t\right)=y_{9}\left(t\right),\;q\left(t\right)=y_{11}\left(t\right),\;{\dot{r}}_{1}\left(t\right)=y_{2}\left(t\right),\;{\dot{r}}_{2}\left(t\right)=y_{4}\left(t\right),\;{\dot{r}}_{3}\left(t\right)=y_{6}\left(t\right),\;{\dot{r}}_{4}\left(t\right)=y_{8}\left(t\right),\;{\dot{r}}_{5}\left(t\right)=y_{10}\left(t\right),\;\dot{q}\left(t\right)=y_{12}\left(t\right).$$

The subject of the solution is then the set of 12 the first order differential equations for unknown functions *y_i_*(*t*), (*i* = 1,2,3,4,5,6,7,8,9,10,11,12) in the form

(55) $${\dot{y}}_{1}\left(t\right)=y_{2}\left(t\right),\;{\dot{y}}_{2}\left(t\right)={\ddot{r}}_{1}\left(t\right),\;{\dot{y}}_{3}\left(t\right)=y_{4}\left(t\right),\;{\dot{y}}_{4}\left(t\right)={\ddot{r}}_{2}\left(t\right),\;{\dot{y}}_{5}\left(t\right)=y_{6}\left(t\right),\;{\dot{y}}_{6}\left(t\right)={\ddot{r}}_{3}\left(t\right),\;{\dot{y}}_{7}\left(t\right)=y_{8}\left(t\right),\;{\dot{y}}_{8}\left(t\right)={\ddot{r}}_{4}\left(t\right),\;{\dot{y}}_{9}\left(t\right)=y_{10}\left(t\right),\;{\dot{y}}_{10}\left(t\right)={\ddot{r}}_{5}\left(t\right),\;{\dot{y}}_{11}\left(t\right)=y_{12}\left(t\right),\;{\dot{y}}_{12}\left(t\right)=\ddot{q}\left(t\right).$$

**Figure 9:**
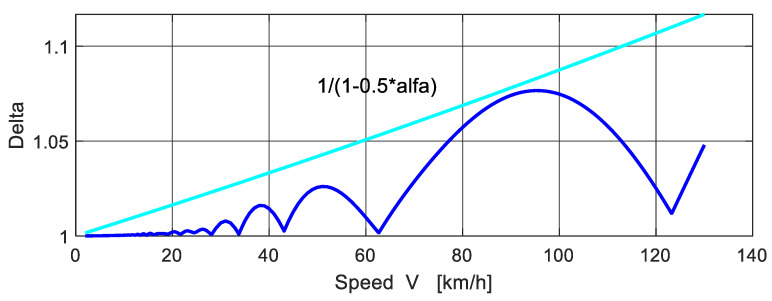
Bridge dynamic factor versus speed of vehicle.

The subject of numerical analysis are the time dependences of dynamic deflections in the middle of the bridge span. As is known, all kinematic quantities of the bridge response depend on the speed of movement of the vehicle [23]. Therefore, in the first step, the dependence of the dynamic factor *δ* of the bridge on the speed of movement of the vehicle was calculated in Figure 9. A smooth bridge surface and zero initial conditions on both the vehicle and the bridge are assumed:

(56) $$r_{i}\left(0\right)={\dot{r}}_{i}\left(0\right)=0,\;i=1,2,3,4,5,\;q\left(0\right)=\dot{q}\left(0\right)=0.$$

The vehicle parameters are:

*m*_1_ = 22,950 kg, *m*_2_ = 2910 kg, *m*_3_ = 2140 kg, *I_y_*_1_ = 62,298 kg·m^2^, *I_y_*_3_ = 932 kg·m^2^,

*k*_1_ = 287,433 N/m, *k*_2_ = 1,522,512 N/m, *k*_3_ = 2,550,600 N/m, *k*_4_ = *k*_5_ = 5,022,720 N/m, 

*b*_1_ = 19,228 kg/s, *b*_2_ = 260,197 kg/s, *b*_3_ = 2746 kg/s, *b*_4_ = *b*_5_ = 5494 kg/s, 

*a* = 3.135 m, *b* = 1.075 m, *c* = 0.66 m, *s* = 4.21 m.

The bridge parameters are:

*L* = 37 m, *μ* = 6442 kg/m, *I* = 0.23186 m^4^, *E*= 1.15e^11^ Pa, *ω_b_* = 0.23321 rad/s.

Maximum static deflection from the vehicle *y_s_* = 9.815068 mm.

**Figure 10:**
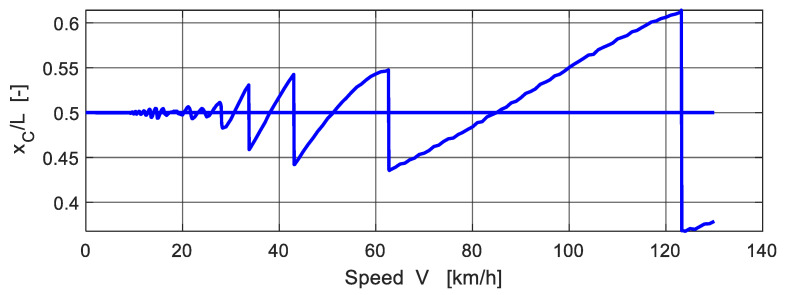
Position of vehicle centrum of gravity on the bridge versus speed of vehicle.

The dependence *δ*(*V*) is a curve that has many local maxima and spikes [24]. The position of the spikes is related to the discontinuous course of the function indicating the position of the vehicle on the bridge at the moment when the maximum deflection occurs. Figure 10 shows the position of the vehicle centre of gravity on the bridge (in dimensionless form *x_C_*/*L*) at the moment when the maximum deflection occurs depending on the speed of the vehicle *V*. For practical purposes, it is appropriate to define an envelope curve, for example in the shape

(57) envelope=1/(1−0.5⋅α)

where *α* is dimensionless speed parameter defined as

(58) $$\alpha =T_{\left(1\right)}/\left(2T_{s}\right).$$

*T*_(1)_ is the period of bridge vibration in the first natural mode and *T_s_* is the time of stay of the vehicle axle on the bridge.

In the second step, the calculation of the time response of the bridge for the characteristic speeds of the vehicle was performed. The speeds 38 km/h, 51 km/h, and 95 km/h correspond to the local maxima of the function *δ*(*V*) and the speeds 43.1 km/h, 62.7 km/h, and 123.2 km/h correspond to the spikes. In this analysis, it was assumed that the vehicle enters the bridge already vibrant. The initial conditions for the vehicle are as follows: 

*r*_1_(0) = −0.02 m, *r*_2_(0) = −0.03 rad, *r*_3_(0) = −0.002 m, *r*_4_(0) = −0.003 m, *r*_5_(0) = 0.0 rad,

${\dot{r}}_{i}\left(0\right)=0,\;i=1,2,3,4,5$.

**Figure 11:**
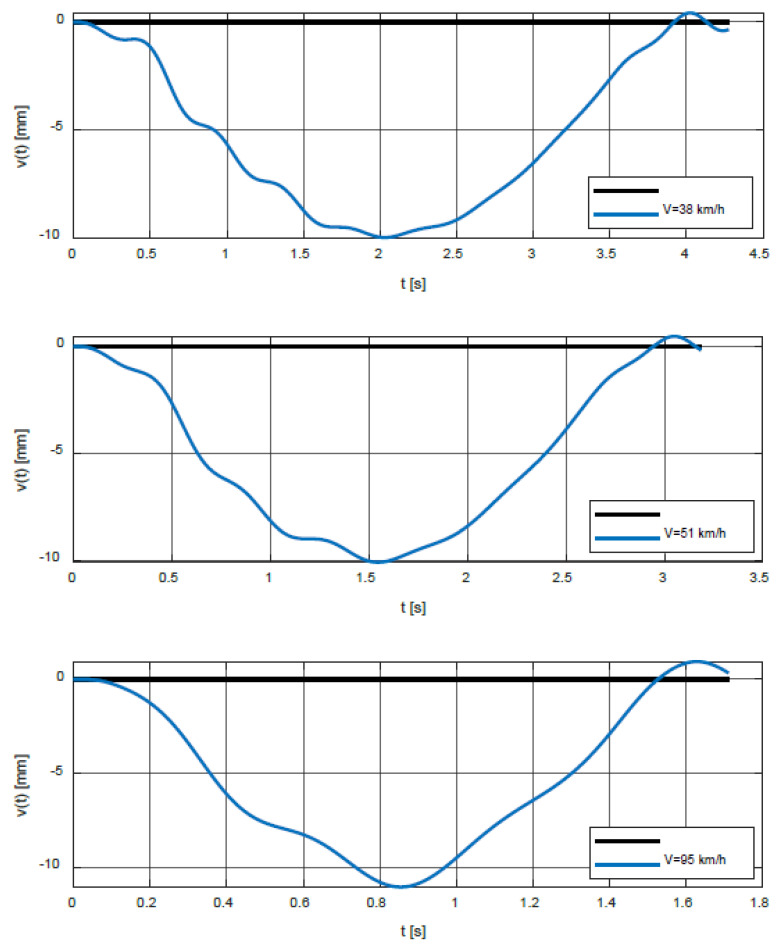
Time courses of the vertical deflections in the middle of the bridge span for speeds 38, 51, and 95 km/h.

**Figure 12:**
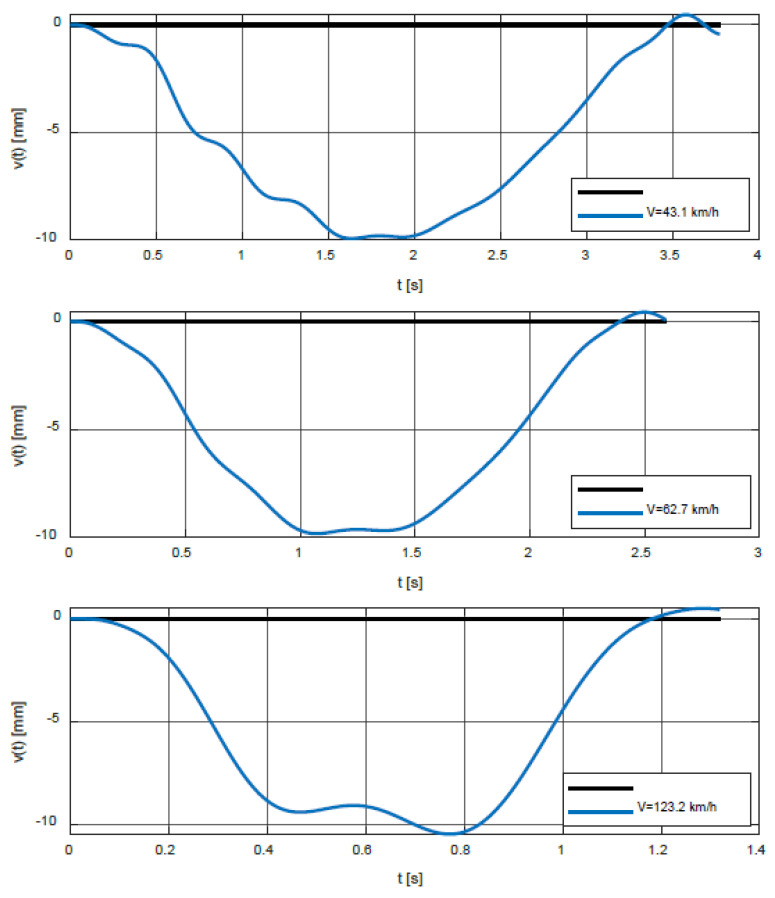
Time courses of the vertical deflections in the middle of the bridge span for speeds 43.1, 62.7, and 123.2 km/h.

Figure 11 shows the time courses of the vertical deflections in the middle of the bridge span for speeds 38, 51, and 95 km/h, and Figure 12 for speeds 43.1, 62.7, and 123.2 km/h.

The maximum bridge deflections and dynamic factors for individual speeds are summarised in Table 2.

## 4. Conclusions

The use of fibre-reinforced polymer composite materials in modern engineering applications has been increasing rapidly. Bridges and aerospace structures are a couple of examples of their application. Steel bridges are replaced by composite materials due to their superior qualities, such as a higher strength-to-weight ratio. Bridge structures are constantly being exposed to various types of loads. The major loads that influence the life of a bridge are dynamic moving loads.

For the forced vibration analysis of laminated polymer composite beams under the effect of moving loads, it is advantageous to use the MATLAB software system. To solve equations of motion, the Runge–Kutta–Fehlberg methods can be used (ode45 solver).

The bridge response to the effects of moving loads is influenced by many factors. The most important of these can be considered the speed of the vehicle. It only makes sense to talk about the influence of all other parameters in connection with the specific speed of the vehicle. If the deflections in the middle of the bridge span are analysed, it is advantageous to work with dimensionless values in the form of dynamic magnification factors δ. The dependence of the dynamic factor on the speed of the vehicle δ(V) is a curve that has many local maxima and spikes. Its shape depends on the type of vehicle used in the analysis. For practical reasons, it is good to define the envelope of amplitudes.

Therefore, in this case, the dynamic magnification factors for the dynamic analysis of composite beams were computed. The maximum dynamic magnification factor occurs at the velocity of 95 km/h.

The ply orientation influences the dynamic behaviour of a beam subjected to a moving load. Based on these results, a designer can choose the right ply orientations to control the dynamic behaviour of laminated beams.

We are still working on research which considers the classic conventional materials, the cross-section of a bridge made of steel girders and a concrete bridge deck. To solve this task of the bridge response to the effects of moving loads due to a heavy truck, a 1D model was adopted. We obtain the maximum difference between the dynamic and static deflection in the middle of the bridge of 21.9%. By replacing conventional materials with laminated composite, we obtained the maximum difference between the dynamic and static deflection in the middle of the bridge of 16.11%. From this point of view, the use of FRP materials in bridge structures is still in its infancy, and there are very clear indications that it will be an excellent choice for a multitude of projects on bridges in the future.

## Figures and Tables

**Table 1:** Material properties of composite laminate.

Property	Value
Mass density of the composite, *ρ*	2100 kg/m^3^
Longitudinal modulus, *E*_1_	214 GPa
Transverse modulus, *E*_2_	18.7 GPa
Longitudinal shear modulus, *G*_12_	4 GPa
Major in-plane Poisson’s ratio, *ν*_12_	0.27
Fibre volume fraction, ξ	0.55
Effective moduli of laminate ((0/90)_25_)_s_, *E_x_ = E_eff_*	115 GPa
Effective shear modulus of laminate ((0/90)_25_)_s_, *G_eff_*	4.8 GPa
Effective in-plane Poisson’s ratio of laminate, *ν_eff_*	0.035

**Table 2:** Maximum bridge deflections and dynamic factors for individual speeds.

Speed *V* (km/h)	*t*_max_. (s)	Max. Dynamic Deflection *v*_max_ (mm)	Dynamic Factor *δ*
38	2.0310	9.9627	1.0150
51	1.5432	10.1018	1.0292
95	0.8544	11.0073	1.1214
43.1	1.6296	9.9250	1.0112
62.7	1.0705	9.8174	1.0002
123.2	0.7711	10.4482	1.0645

## Data Availability

The data presented in this study are available on request from the corresponding authors.
